# Silexan reduces functional impairment in patients with a major depressive episode

**DOI:** 10.1192/j.eurpsy.2025.856

**Published:** 2025-08-26

**Authors:** H.-P. Volz, S. Klement, E. Seifritz, S. Kasper

**Affiliations:** 1 Medical Advisor, Würzburg; 2 Dr. Willmar Schwabe GmbH & Co. KG, Karlsruhe, Germany; 3 Psychiatric Hospital, Zürich, Switzerland; 4 Medical University of Vienna, Vienna, Austria

## Abstract

**Introduction:**

In a multi-centre, double-blind, randomised, placebo- and reference-controlled clinical Phase III trial in patients with a mild to moderate major depressive episode, Silexan, a special essential oil from *Lavandula angustifolia,* demonstrated a clinically meaningful and statistically significant antidepressant effect (Kasper *et al*. Eur Arch Psychiatry Clin Neurosci 2024; Apr 1, Epub ahead of print). Patients with depression often experience functional impairment, i.e. in their ability to perform activities of daily living, in their capacity to form and maintain interpersonal relationships, and in their work productivity.

**Objectives:**

We present data from the trial to assess the impact of the symptoms of the depressive episode on the patient`s functional abilities in daily living.

**Methods:**

Adult patients (≥18 years) suffering from a major depressive episode of mild to moderate severity according to ICD‑10 were included. Further inclusion criterion was a total score of 19 – 34 points in the Montgomery-Åsberg-Depression Rating Scale (MADRS). Randomised patients took 80 mg Silexan, 50 mg Sertraline, or placebo once daily over 8 weeks. Functional impairment was assessed using the change of the Sheehan Disability Scale (SDS total score) between baseline and week 8, changes of each SDS single item “work” (item 1), “social life/leisure activities” (item 2), and “family life/home responsibilities” (item 3), as well as the number of lost (DL) and underproductive days (DU). The scientific questions if the active treatment groups are superior to placebo in improving functional impairment was investigated as secondary objectives, which were analysed descriptively using an analysis of covariance model with factors for treatment and centre and the baseline value as covariate.

**Results:**

The full analysis set consisted of 498 patients. The changes of the SDS total score differed by 2.40 (p<0.001) points (adjusted mean difference) in favour of Silexan compared to placebo and by 0.82 (p=0.267) points in favour of Sertraline compared to placebo. The mean differences between Silexan and placebo were 0.98 points for item 1, 0.65 for item 2, and 0.78 for item 3 (p<0.05, each). Compared to placebo, both DL (p=0.055) and DU (p=0.011) occurred less frequently with Silexan. In the primary efficacy endpoint of the trial, the change of the MADRS total score, Silexan was significantly superior to placebo (p<0.01).

**Conclusions:**

With Silexan treatment, patients with a major depressive episode coped better with work, social life, or family responsibilities and had fewer lost or underproductive days.

**Disclosure of Interest:**

H.-P. Volz Consultant of: Lundbeck, Pfizer, Schwabe (Spitzner), Bayer, Janssen, neuraxpharm, AstraZeneca, S. Klement Employee of: Dr. Willmar Schwabe GmbH & Co. KG, E. Seifritz Grant / Research support from: Swiss National Science Foundation and University of Zürich, Consultant of: Lundbeck, Schwabe, Janssen, Otsuka, Mepha Pharma, Otsuka Pharma, Recordati, OM Pharma, Takeda, Sunovion Pharma and Angelini, S. Kasper Consultant of: Angelini, Biogen, Boehringer, Esai, Janssen, IQVIA, Mylan, Recordati, Rovi, Sage and Schwabe, Speakers bureau of: Angelini, Aspen Farmaceutica S.A., Biogen, Janssen, Recordati, Schwabe, Servier, Sothema, and Sun Pharma

